# Neonatal seizures: Case definition & guidelines for data collection, analysis, and presentation of immunization safety data

**DOI:** 10.1016/j.vaccine.2019.05.031

**Published:** 2019-12-10

**Authors:** Serena Pellegrin, Flor M. Munoz, Michael Padula, Paul T. Heath, Lee Meller, Karina Top, Jo Wilmshurst, Max Wiznitzer, Manoja Kumar Das, Cecil D. Hahn, Merita Kucuku, James Oleske, Kollencheri Puthenveettil Vinayan, Elissa Yozawitz, Satinder Aneja, Niranjan Bhat, Geraldine Boylan, Sanie Sesay, Anju Shrestha, Janet S. Soul, Beckie Tagbo, Jyoti Joshi, Aung Soe, Helena C. Maltezou, Jane Gidudu, Sonali Kochhar, Ronit M. Pressler, for the Brighton Collaboration Neonatal Seizures Working Group

**Affiliations:** a Clinical Neuroscience, UCL-Institute of Child Health, London, UK; b Department of Child Neuropsychiatry, University of Verona, Verona, Italy; c Baylor College of Medicine, Department of Pediatrics, Houston, TX, USA; d The Children’s Hospital of Philadelphia, PA, USA; e Vaccine Institute, St Georges University of London, London, UK; f Syneos Health, Safety & Pharmacovigilance, Raleigh, NC, USA; g Department of Pediatrics, Dalhousie University, Halifax, NS, Canada; h Department of Paediatric Neurology, Red Cross War Memorial Children’s Hospital, Neuroscience Institute, University of Cape Town, South Africa; i Rainbow Babies & Children’s Hospital, Cleveland, OH, USA; j The INCLEN Trust International, New Delhi, India; k Division of Neurology, The Hospital for Sick Children and Department of Paediatrics, University of Toronto, Toronto, Canada; l National Agency for Medicines and Medical Devices, Tirana, Albania; m Department of Pediatrics, Rutgers – New Jersey Medical School, Newark, NJ, USA; n Division of Pediatric Neurology, Department of Neurology, Amrita Institute of Medical Sciences, Cochin, Kerala, India; o Saul R. Korey Department of Neurology, Department of Pediatrics, Albert Einstein College of Medicine, Bronx, NY, USA; p Department of Pediatrics, School of Medical Sciences & Research, Sharda University, Gr Noida, India; q Center for Vaccine Innovation and Access PATH, Seattle, WA, USA; r INFANT Research Centre, University College Cork, Ireland; s Clinical Sciences, Sanofi Pasteur, Marcy L’Etoile, France; t Sanofi Pasteur, Global Pharmacovigilance, PA, USA; u Department of Neurology, Boston Children’s Hospital, Harvard Medical School, Boston, MA, USA; v Institute of Child Health, University of Nigeria Teaching Hospital, Nigeria; w Center for Disease Dynamics, Economics & Policy, New Delhi, India; x Medway NHS Foundation Trust, Kent, UK; y Department for Interventions in Healthcare Facilities, Hellenic Center for Disease Control and Prevention, Athens, Greece; z Centers for Disease Control and Prevention, Global Immunization Division, Atlanta, USA; aa Global Healthcare Consulting, New Delhi, India; ab Department of Public Health, Erasmus MC, University Medical Center, Rotterdam, the Netherlands; ac Department of Global Health, University of Washington, Seattle, USA; ad Great Ormond Street Hospital for Children NHS Foundation Trust, London, UK

**Keywords:** Neonatal seizures, Adverse event, Immunization, Guidelines, Case definition, ACNS, American Clinical Neurophysiology Society, aEEG, amplitude-integrated EEG, BCG, bacille Calmette-Guérin, cEEG, conventional EEG, EEG, electroencephalography, GA, gestational age, HBW, high birth weight (≥4000 g), ILAE, International League Against Epilepsy, LBW, low birth weight (≥1500–2499 g), MRI, Magnetic Resonance Imaging, NBW, normal birth weight (≥2500–3999 g), NICU, neonatal intensive care unit, PMA, post menstrual age, Tdap, tetanus-diphtheria-acellular pertussis, Tp, tetanus-diphtheria-acellular pertussis, VLBW, very low birth weight (<1500 g), WHO, World Health Organization

## 1 Preamble

### 1.1 Need for developing case definitions and guidelines for data collection, analysis, and presentation for neonatal seizures as an adverse event following immunization

Seizures are the most common neurological emergency in newborns and can be associated with significant mortality and neuro-developmental disability. Neonatal seizures are a major challenge for clinicians because of inconspicuous clinical presentation, variable electro-clinical correlation, and poor response to antiseizure drugs. It is well recognized that fever and infection can trigger seizures in young children and that this risk is enhanced in children with epilepsy. As immunization may cause a fever, vaccination can be a non-specific trigger for seizures in children [1]. On the other hand, children with epilepsy do not appear to be at increased risk of seizures following immunization [2]. It is unclear whether vaccination in newborns or maternal vaccination, is associated with a higher risk of neonatal seizures. However, as maternal immunization with established vaccines becomes more prevalent across multiple geographies, and new maternal vaccine candidates enter late-stage development, it is becoming increasingly important to create easily adopted standard definitions for adverse events potentially associated with these interventions. The Brighton Collaboration has previously published a case definition for seizures in children [3] but not for seizures in neonates.

#### 1.1.1 Epidemiology of neonatal seizures

The reported prevalence and incidence of neonatal seizures vary considerably due to differences in study methodology, especially in the identification of neonatal seizures, and geographic setting [4], [5]. The majority of seizures in neonates present without clinical signs [6], [7] and can be recognized only with cEEG (conventional electroencephalography) monitoring, which has not been used in all studies. Therefore, the exact incidence of electrographic, clinically silent neonatal seizures in term and preterm babies is not known (Table 1, Table 2).

**Table 1:** Incidence of neonatal seizures.

Area	Setting	Population	Seizure detection	Incidence	Ref.
USA	NICU (1985–89)	Term and preterm (n = 16,428)	Clinical/EEG (Record review)	Overall: 3.5/1000 live births VLBW: 57.5/1000 live births LBW: 4.4/1000 NBW: 2.8/1000 live births HBW: 2.0/1000 live births	[8]
USA	NICU (1992–94)	Term and preterm (n = 116,048)	Clinical (Record review)	Overall: 1.8/1000 live births VLBW: 19/1000 live births	[9]
Canada	NICU (1990–95)	Term and preterm	Clinical/cEEG	Overall: 2.5/1000 live births	[10]
UK	NICU (2007–08)	Preterm (<30 weeks) (n = 51)	aEEG	22% (aEEG) 4% (clinically)	[11]
India	NICU (2011–13)	Term and preterm (n = 10724)	Clinical	1.6% clinical seizure in first 28 days	[12]
Iran	NICU (2007–09)	Term and preterm (n = 699)	Clinical	3.6% of NICU admission 5/1000 live births (extrapolated)	[13]
Iran	NICU (2008–11)	Term and preterm (n = 1112)	Clinical	9.1% of NICU admission	[14]
Kenya	NICU (2003–07)	Term and preterm (n = 1600)	Clinical	9% of NICU admissions 39.5/1000 live births (extrapolated)	[15]

xLegend: NICU (neonatal intensive care unit), VLBW (<1500 g), LBW (≥1500–2499 g), NBW (≥2500–3999 g), HBW (≥4000 g), cEEG (conventional EEG), aEEG (amplitude-integrated EEG).

**Table 2:** Etiology of neonatal seizures and reported relative frequency in high-, middle- and low- income countries.

Etiology	High-income countries [8], [10], [16], [23], [24], [25], [26]	Middle and Low- income countries [12], [13], [23], [24], [27], [28], [29], [30]	Pooled [5], [27], [28]
Hypoxic-ischemic encephalopathy	38–46%	8–77.9%	12.5–77%
Intracranial hemorrhage	12%	6.9–26%	7–17%
Cerebral infarction	7–18%	12.8%	6–17%
Cerebral malformations	2.9–10%	1.1–5.2%	3–17%
Infections	4–20%	8–60%	0.7–24%
Metabolic			
– Hypoglycemia	4–9%	1–16.2%	1–13%
– Electrolytes (Na, K, Ca, Mg)	6%	2.8–14.9%	0.5–43%
– Inborn errors of metabolism	3%	1–2.1%	3–4%
Hyperbilirubinemia/kernicterus	N/A	4.6–12%	1%
Maternal drug withdrawal	N/A	1.7%	4%
Genetic	3–6%	N/A	N/A
Unknown	9–14%	2.1%	2%

*Incidence.* The reported incidence of neonatal seizures worldwide varies from 1.0–4.4 per 1000 livebirths in high-income countries (USA) [8], [9], [16], to 5 per 1000 live births in upper middle-income countries (Iran) [13]. Reports from low- and middle-income countries are limited, but one study from Kenya reported an incidence of 39.5 per 1000 live births [15]. Among the preterm population, incidences vary considerably according to different methods of diagnosis. Based only on clinical observation the incidence of seizure in preterms has been reported to be 3.9–57.5 per 1000 live births [8], [10], [17], whereas studies using amplitude-integrated electroencephalography (aEEG), reveal a seizure burden up to 48% [11], [18], [19]. However, it is well recognized that aEEG can be falsely positive particularly in preterm infants [20]. Studies using cEEG in preterms indicate an incidence of 4–9% in high-income countries (75% of which are electrographic-only seizures) [21], [22].

#### 1.1.2 Etiology of neonatal seizures

The etiology of neonatal seizures is heterogeneous, and sometimes unknown, although the majority are due to hypoxia-ischemia, stroke or infections in term infants. In preterm infants, intraventricular hemorrhage is the commonest cause of seizure [29], [30].

The heterogeneity in the etiologic profile of neonatal seizures across geographies and economic strata is due to two main factors: differences in obstetric/perinatal care and access to electrodiagnostic techniques leading to differing rates of detection and diagnosis (Table 2).

#### 1.1.3 Timing of onset

The onset of neonatal seizures depends on etiology and is most common within the first week of life, with 25–55% occurring in the first 24 h [15], [24], [31]. Onset is generally later in preterm compared to term infants [29].

#### 1.1.4 Risk factors

Maternal risk factors for neonatal seizures include maternal age >40 years, nulliparous, diabetes mellitus, chorioamnionitis, traumatic delivery, prolonged second stage of labor, fetal distress, placental abruption, cord prolapse, and uterine rupture[23].

Neonatal risk factors for seizures include the etiologies for seizure listed in Table 2.

#### 1.1.5 Outcomes

While a normal neurological outcome after neonatal seizures is reported in 25–40% of infants [21], [32], 15–30% develop cerebral palsy [32], [33], [34]; 30–50% developmental delay [21], [32]; and 20–35% epilepsy [32], [33]. The prognosis of neonatal seizures depends on the underlying etiology. However, there is evidence that seizures are independently associated with worse outcome [35], [36]. Risk factors identified for poor outcome following neonatal seizures include prematurity/low birth weight, severity of HIE, high-grade intraventricular hemorrhage, persistently abnormal EEG background activity, seizure burden (electrographic seizure burden of >13 min/h), presence of neonatal status epilepticus (but not recurrent seizures), central nervous system infection and cerebral dysgenesis [4], [26], [35], [37], [38]. Death is reported among 7–25% of neonates with seizures in low-, middle-, and high-income countries [15], [25], [32], [36], mostly due to the underlying etiology. Mortality is higher among preterm and low-birthweight neonates (30–33%) [22], [39].

#### 1.1.6 Pathophysiology of neonatal seizures

Developmental age-specific mechanisms influence the generation and phenotype of seizures. While there are some limitations in the use of animal models to study neonatal seizures, conclusions can be reached with consideration of the species-specific maturation rates in the system of interest [40].

The neonatal period is a time of intense brain development. While cortical lamination is fully developed in the term infant, neurite outgrowth and synaptogenesis are continuing and are in their elementary stages. Brain myelination is immature. These factors limit the rapid propagation of neonatal seizures and their clinical presentation (with generalized, from onset, tonic-clonic seizures rarely occurring) [41].

In the neonatal brain, the balance between excitatory versus inhibitory synapses is tipped in favor of excitation to permit robust activity-dependent synaptic formation, plasticity, and remodeling. Glutamate is the major excitatory neurotransmitter in the CNS with the involvement of AMPA and NMDA receptors and more expression and function than in the adult brain. For example, while, in the adult brain, γ-amino-butyric acid (GABA) usually induces membrane hyperpolarization, early in the developing brain it induces membrane depolarization by causing Cl¯ efflux rather than influx. The HCN channels, which are members of the K^+^ channel super-family and important for maintenance of resting membrane potential and dendritic excitability, are also developmentally regulated. The immature brain has relatively low expression of the HCN1 isoform, which serves to reduce dendritic excitability in the adult brain [40].

Genetic epilepsies with onset in the neonatal period reflect the structural and physiologic factors that can lead to neonatal seizures. These include ion channel function (e.g. KCNQ2), excitation-inhibition balance (e.g. pyridoxine-dependent epilepsy), brain development (e.g. ARX) and synaptic function (e.g. STXBP1) [42]. Some of the epilepsy syndromes with neonatal seizures have a favorable or “benign” prognosis (self-limiting familial neonatal seizures), however there exist severe epileptic encephalopathies with a poor outcome (neonatal myoclonic encephalopathy and early infantile epileptic encephalopathy or Ohtahara syndrome).

#### 1.1.7 Diagnosis of neonatal seizures

The clinical diagnosis of neonatal seizures is challenging because many neonatal seizures either manifest with subtle clinical signs or remain entirely subclinical despite the presence of clear electrographic seizure activity on EEG.

Clinical manifestations of neonatal seizures may include focal motor movements or non-motor signs [79], but manifestations are usually discreet and are often difficult to distinguish from other physiologic non-seizure movements such as eye deviation, automatisms, apnea and limb posturing [43]. Furthermore, numerous studies applying conventional EEG (cEEG) monitoring in neonatal cohorts have consistently demonstrated that the majority of neonatal seizures are subclinical [7], [44], especially in preterm infants [45].

The diagnosis of neonatal seizures may be made by cEEG, amplitude-integrated EEG (aEEG) or by clinical signs alone. Gold-standard is capturing a seizure on cEEG (ictal EEG) because it provides the most direct and comprehensive assessment of neuronal activity. In comparison, aEEG is less accurate because it employs fewer electrodes over a smaller spatial area and the aEEG display is filtered and time-compressed making it harder to identify brief seizures. When aEEG is used together with a real-time EEG channel, the median sensitivity for seizure identification is 76% (range: 71–85%), and the median specificity is 85% (range: 39–96%). When aEEG was used without a real-time EEG channel, the median sensitivity is 39% (range: 25–80), and specificity is 95% (range 50–100) [46]. On the other hand, when the goal is identifying only the presence or absence of seizures in a neonate rather than individual seizures, the median sensitivity of aEEG with a real-time EEG channel rises to 85% (range: 70–90%).

Among neonates who present with clinically apparent seizures, antiseizure drugs commonly suppress clinical activity, but ongoing electrographic seizures persist, a phenomenon termed uncoupling [47], [48], [49], [50]. Because of this uncoupling, which can also occur spontaneously, aEEG or cEEG monitoring is even more essential for the accurate assessment of response to therapy and seizure burden [51]. Practitioners should be aware of the limitations of the clinical assessment in over and under-diagnosing seizures, and aEEG or cEEG confirmation of clinically-diagnosed seizures should be sought whenever possible.

#### 1.1.8 Differential diagnosis

Early recognition and accurate diagnosis of seizures in the neonatal period is essential for optimal management. However, the clinical diagnosis of seizures in neonates is also challenging because infants may present with abnormal movements that are non-epileptic but are mistaken for seizures leading to inappropriate treatment and unwarranted prognostic concern [52]. While the most common non-epileptic movements are generally benign and associated with a good prognosis, some may be associated with pathologic conditions. The video-EEG recording of the event can be very helpful to differentiate seizure from non-epileptic events. Seizures can coexist with non-epileptic manifestation in some patients. Table 3 summarizes the characteristics of the most common non-epileptic manifestation in newborns.

**Table 3:** Differential diagnosis of neonatal seizures.

Syndrome	Etiology	Description of events	Prognosis/outcome	Ref.
Jitteriness/tremor	Physiological, or secondary (HIE, metabolic, etc.)	Tremors (rhythmical oscillatory movements), stimulus sensitive, diminish with passive flexion of extremity	Dependent on cause	[53], [54]
Benign neonatal sleep myoclonus		Sudden involuntary jerking with a higher amplitude than tremor, that occur solely during sleep	Excellent	[55], [56]
Startle disease (hyperekplexia)	Genetic, autosomal dominant	Exaggerated startle response may present with apnea and severe spasms	Stiffness resolves by three years, exaggerated startle remains	[52], [54]
Paroxysmal extreme pain disorder	Genetic, autosomal dominant	May present with flushing, tonic spasms, bradycardia, and syncope	Paroxysmal episodes of deep burning pain	[52]
Acute bilirubin encephalopathy	Unconjugated hyperbilirubinemia	May present with acute neurologic signs such as hypertonia, oculogyric movements and dystonic posturing	Depending on levels	[57], [54]
Neonatal tetanus	Exposure to spores of Clostridium tetani	Muscle spasms and severe rigidity may present with poor feeding due to trismus	Mostly fatal	[58]
Autonomic paroxysms		Episodes of apnea, pallor, flushing, and cyclic periods of tachycardia or hypertension		[59], [54]
Sandifer syndrome	Gastroesophageal reflux	Episodic dystonic posturing with torticollis and severe hyperextension (opisthotonos)	Usually good	[60], [61]
Tonic posturing	Severe hypoxic brain injury	Generalized tonic posturing	Poor	[28], [62]
Other non-epileptic myoclonus		Benzodiazepine exposure in preterm infant, infants of opiate dependent mothers	Related to underlying cause	[54], [55]

#### 1.1.9 Neonatal seizures following maternal or neonatal vaccination

*Maternal vaccination.* A literature search conducted by the authors did not identify any reports of seizures among newborns born to women who received tetanus-diphtheria-acellular pertussis (Tdap), tetanus toxoid, tetanus-diphtheria (Td), seasonal or pandemic influenza vaccines, or in randomized controlled trials of investigational Group B *Streptococcus* or respiratory syncytial virus vaccines. A retrospective cohort study of pertussis among infants <63 days of age reported no seizures among 34 infants (median age 45 days) whose mothers received Tdap during pregnancy, while 14/336 (4%) infants of unvaccinated mothers developed seizures with pertussis infection (relative risk 0.96; 95% CI 0.94–0.98) [63]. There is currently no evidence of an association between vaccination during pregnancy and neonatal seizures.

*Neonatal vaccination.* In a study of claims in the United States National Vaccine Injury Compensation Program of seizures and/or encephalopathy allegedly caused by an immunization among children younger than two years during 1995–2005, a total of 90 claims (60%) concerned babies between 0 and 6 months of age but the number of neonates was not reported [64]. In 12 cases (7.2%) the final diagnostic impression by a pediatric neurologist was “infantile seizures”. This article provides no certainty about a causal effect because it is a summary of individual cases in a litigation setting. Another study found no increase in seizures or other neurologic events among healthy, full-term neonates who received hepatitis B vaccination versus controls [65]. In addition, there were no reports of neonatal seizures after polio or bacille Calmette-Guérin (BCG) vaccination, the vaccinations most commonly used in the neonatal period [66].

#### 1.1.10 Existing definitions for neonatal seizures

Several definitions of neonatal seizures exist (Table 4). Neonatal seizures are traditionally defined as paroxysmal alterations in neurologic function (including motor, behavior and/or autonomic function) occurring in the first 28 days after birth of a term neonate or before 44 weeks of gestational age in a preterm infant [67]. It should be noted that this purely clinical definition of neonatal seizures is entirely arbitrary, resulting in both over and underestimation of the number of seizures in the newborn [7]. Several studies have shown the existence of considerable inter-observer variability among physicians and allied health professionals in the clinical diagnosis of seizures in the NICU [68]. According to the International League Against Epilepsy (ILAE), an epileptic seizure is defined as an electro-clinical phenomenon characterized by the transient occurrence of signs and symptoms due to an abnormal, excessive or synchronous neuronal activity in the brain [69]. Therefore, the identification of ictal discharges on the EEG (electrographic seizure) should be considered the gold standard for the accurate diagnosis of neonatal seizures (see Section 1.1.7). A recent World Health Organization’s (WHO) guideline on neonatal seizures also recommended the use of EEG for the confirmation of suspected neonatal seizures at all levels of care [27].

**Table 4:** Existing definitions of neonatal seizures.

References [#]	Definition of Neonatal seizure
Clancy et al., 1987 [70]	An **electrographic seizure** is defined as a clear ictal event characterized by the appearance of sudden, repetitive, evolving stereotyped waveforms with a definite beginning, middle, and end; lasting an (arbitrary) minimum ictal duration of 10 s
Volpe, 1989 [71]	A seizure is defined **clinically** as a paroxysmal alteration in neurologic function, i.e., behavioral, motor, and/or autonomic function. Such a definition includes clinical phenomena that are associated temporally with (surface-recorded) EEG seizure activity and therefore are clearly epileptic, i.e., related to hypersynchronous electrical discharges that may spare and activate other brain structures. The definition also includes paroxysmal clinical phenomena that often are not associated temporally with EEG seizure activity; whether any of these clinical phenomena may also be epileptic (e.g. related to hypersynchronous electrical discharges from subcortical structures and not detected by surface EEG) is not entirely clear
ILAE, Fisher et al., 2005 [69]*	An **epileptic seizure** is a transient occurrence of signs and/or symptoms due to abnormal excessive or synchronous neuronal activity in the brain
Andre et al., 2010 [72]	**Critical or ictal discharges** are abrupt and transient changes in background activity; their duration ranges from 10 s to several minutes
ACNS, Tsuchida et al., 2013 [73]	An **electrographic seizure** is a sudden, abnormal EEG event defined by a repetitive and evolving pattern with a minimum 2 mV pp voltage and duration of at least 10 s. A seizure is always an abnormal pattern and should not be confused with transient background changes, such as those associated with drowsiness or arousal from sleep. “Evolving” is defined as an unequivocal evolution in frequency, voltage, morphology, or location

Legend: ILAE (International League Against Epilepsy); ACNS (American Clinical Neurophysiology Society).

* Not specifically for neonatal seizure

#### 1.1.11 Classification of neonatal seizures

Neonatal seizures are focal, often subclinical [6] or have discreet clinical manifestations that are difficult to differentiate from movements of severely ill newborns [71], [74]. Historically, seizure semiology in the neonatal period was considered to differ to those of other ages and therefore specific classification systems for neonates were developed. Some classification systems are based on direct observation only [71], [75], [76], [77], whereas others are based on clinical observation and video EEG [74] (Table 5). However, there is no universally accepted classification in the neonatal period and therefore no common language to describe neonatal seizures. The 2017 ILAE Position Papers on Classification [77], [78] are important updates on the terminology and etiology of seizures but specifically do not include neonatal seizures. A Neonatal Seizure Task Force of the ILAE has proposed a new framework that uses EEG and clinical seizure semiology to classify seizures in the neonatal period according to the predominant seizure type (electrographic only, motor, or non-motor) [79]. Motor seizures may be automatisms, clonic, epileptic spasms, myoclonic, sequential or tonic and non-motor seizures may be autonomic or behavior arrest seizures.

**Table 5:** Classifications used for neonatal seizures.

Reference [#]	Target group (age)	EEG diagnostic criteria	Electrographic seizures	Use of ILAE terminology
Volpe, 1973, 1989 [71], [80]	Neonates	No	No	No
Mizrahi & Kellaway, 1987 [74]	Neonates	Yes	Yes	Partially
ILAE, 1981*[76]	>1 month	No	No	Yes
ILAE, Fisher et al., 2017*[77]	>1 month	No	No	Yes
ILAE, Pressler et al. [79]	Neonates	Yes	Yes	Yes

Legend: ILAE (International League Against Epilepsy).

* Not specifically for neonatal seizure.

#### 1.1.12 Need for a harmonized definition of neonatal seizures in the neonate

There is no uniformly accepted definition of neonatal seizures. This provides the opportunity to offer a definition that is practical and useful in the context of neonatal seizures following maternal and neonatal immunization, as data comparability across trials or surveillance systems will facilitate data interpretation and the assessment of vaccine safety, as well as promote the scientific understanding of neonatal seizures.

### 1.2 Methods for the development of the case definition and guidelines for data collection, analysis, and presentation for neonatal seizures as an adverse events following immunization

Following the process described in the overview papers [81], [82] as well as on the Brighton Collaboration Website http://www.brightoncollaboration.org/internet/en/index/process.html, the Brighton Collaboration *Neonatal Seizures Working Group* was formed in 2018 and included members with clinical, academic, public health, industry backgrounds.

To guide the decision-making for the case definition and guidelines, we conducted a literature search using Medline, Embase and the Cochrane Central Register for English language articles reporting on seizures among neonates born to women vaccinated during pregnancy. In addition, we searched for clinical trials, passive and active surveillance reports, cohort and case-control studies of specific vaccines evaluated in pregnancy to capture additional reports of neonatal seizures and confirm the findings of our primary literature review. Only English language articles and articles referring to humans were selected for review. The primary search identified 82 articles excluding duplications of which 80 were excluded based on review of the title of abstract. The remaining two articles were excluded after review of the full text as they did not provide information regarding neonatal seizures and vaccines. A search for adverse events after maternal Tdap vaccination identified one relevant article that mentioned neonatal seizures.

We extended the search to include reports of neonates with seizure after immunization at birth, following the same methods described above. A total of 194 articles excluding duplications were identified. Based on abstract content we selected 12 articles for complete reading. Articles were excluded mainly because they presented no detailed information about the age of the vaccinated infants (e.g. “infants 0–6 months”) or the specific vaccination schedule. Finally, only one original article was selected for inclusion in our systematic review [65].

### 1.3 Rationale for selected decisions about the case definition of neonatal seizures as an adverse event following immunization

The working group agreed that electrographically documented seizures with or without clinical manifestations represent the most accurate concept of neonatal seizures. There are several operational definitions for electrographic seizures in the newborn. According to the American Clinical Neurophysiology Society (ACNS), an electrographic seizure in a newborn is defined as a sudden, abnormal EEG event characterized by a rhythmic and evolving pattern with a minimum 2 µV peak-to-peak voltage and duration of at least 10 s. “Evolving” is defined as an unequivocal evolution in frequency, voltage, morphology, or location [73]. However, the working group considered at length the operational difficulties of a purely electrographic definition. The cut-off of 10 s of duration is arbitrary and does not include shorter clinical seizures e.g. myoclonic jerks or spasms. Prolonged EEG monitoring in the NICU on critically ill term/preterm newborns with multiple hemodynamic supports may be technically very demanding and may not be easily available in many centers, even in high-income countries. Another limiting factor will be the non-availability of adequate and appropriately trained personnel with special expertise in the recording and interpretation of EEG in the neonatal ICU setting.

Amplitude-integrated EEG (aEEG) can be a useful instrument but less accurate (see Section 1.1.7 for further details).

Clinical diagnosis of neonatal seizures is the least accurate parameter, although some clinical manifestations, such as focal clonic seizures or focal tonic seizures, particularly when seizures are stereotyped and recurrent, are highly indicative of epileptic seizures [68]. In contrast, events with generalized tonic posturing seen in infants with diffuse severe brain injury are usually of non-epiletic origin [28].

#### 1.3.1 Related terms of neonatal seizures

**Neonatal period:** begins at birth and ends at 28 completed days of life [83].

**Gestational age (GA):** is a clinical term that applies to the estimated age of the fetus during pregnancy, generally given in weeks and days from the first day of the last menstrual period. According to the International Statistical Classification of Diseases and Related Health Problems (ICD-10) [84], GA is used to classify three different periods in relation to delivery: preterm births (less than 37 weeks), term births (37–41 weeks) and post-term births (42 weeks or more). For additional information refer to the premature birth Case Definition of the Brighton Collaboration Preterm Birth Working Group [85].

**Neonatal seizures:** relate to epileptic seizures in the neonatal period. It includes terms such as neonatal convulsions, neonatal fits, neonatal epilepsy and neonatal convulsive disorder (the latter two refer to a disorder with repeated unprovoked epileptic seizures, see below). The preferred term is neonatal seizure.

**Epilepsy** refers to a disorder with at least two unprovoked (or reflex) seizures occurring greater than 24 h apart or one unprovoked (or reflex) seizure and a probability of further seizures similar to the general recurrence risk (at least 60%) after two unprovoked seizures, occurring over the next 10 years [86].

#### 1.3.2 Focus of Brighton Collaboration case definition

The focus of the working group was to agree on a harmonized definition of neonatal seizures and the criteria to identify them, with different levels of diagnostic certainty. This will be useful also for the identification of neonatal seizures in the context of vaccination of mothers during pregnancy or neonatal vaccination.

#### 1.3.3 Formulating a case definition that reflects diagnostic certainty: weighing specificity versus sensitivity

It needs to be emphasized that the grading of definition levels is entirely about diagnostic certainty, not the clinical severity of an event. Thus, a very severe clinical event may appropriately be classified as possible (level 3) or probable (level 2), rather than definite (level 1), if it could reasonably be of a non-epileptic etiology. Detailed information about the severity of the event should additionally always be recorded, as specified by the data collection guidelines.

The number of symptoms and/or signs that will be documented for each case may vary considerably. The case definition has been formulated such that the level 1 definition is highly specific for the condition. As maximum specificity normally implies a loss of sensitivity, two additional diagnostic levels have been included in the definition, offering a stepwise increase of sensitivity from level 1 down to level 3, while retaining an acceptable level of specificity at all levels. In this way, it is hoped that all possible cases of neonatal seizures can be captured.

#### 1.3.4 Rationale for individual criteria or decision made related to the case definition

The working group agreed to a definition of neonatal seizures (see below) and to give different levels of certainty in the diagnosis (depending on the use of instrumental tools such as cEEG and aEEG or the sole clinical observation) in order to be effective and applicable in high-, middle- and low-income countries.

Pathology, radiology and laboratory findings are not included in the case definition, although they can provide important information regarding the causes of neonatal seizure.

#### 1.3.5 Influence of treatment on the fulfilment of the case definition

The working group decided against using “treatment” or “treatment response” towards the fulfillment of the case definition of neonatal seizures.

A treatment response or failure is not in itself diagnostic, as less than 50% of neonatal seizures respond to the first line treatment (phenobarbital) [27], [87], [88]. At the same time, many antiseizure drugs have sedative or central nervous system depressant effects and may reduce the intensity or frequency of non-epileptic movements. It is only in certain circumstances, such as acute symptomatic seizures due to hypoglycemia or pyridoxine-dependent seizures, that specific treatments have diagnostic implications.

#### 1.3.6 Timing post maternal immunization

Specific time-frames for the onset of symptoms of neonatal seizures following maternal immunization are not included. No information is available regarding the potential relevance of the timing of maternal immunization and the occurrence of neonatal seizures.

We postulate that a definition designed to be a suitable tool for testing causal relationships requires ascertainment of the outcome (e.g. neonatal seizures) independent from the exposure (e.g. maternal immunization). Therefore, to avoid selection bias, a restrictive time interval from maternal immunization to onset of neonatal seizures should not be an integral part of such a definition. Instead, where feasible, details of this interval should be assessed and reported as described in the data collection guidelines.

Furthermore, neonatal seizures often occur outside the controlled setting of a clinical trial or hospital. In some settings, it may be impossible to obtain a clear timeline of the event, particularly in low resource and rural settings. To avoid exclusion of such cases, this Brighton Collaboration case definition avoids setting arbitrary time-frames between maternal immunization and occurrence of the defined event.

### 1.4 Guidelines for data collection, analysis and presentation

As mentioned in the overview, the case definition is accompanied by guidelines which are structured according to the steps of conducting a clinical trial, i.e. data collection, analysis and presentation. Neither case definition nor guidelines are intended to guide or establish criteria for management of ill infants, children, or adults. Both were developed to improve data comparability.

### 1.5 Periodic review

Similar to all Brighton Collaboration case definitions and guidelines, review of the definition with its guidelines is planned on a regular basis (i.e. every three to five years) or more often if needed.

## 2 Case definition of neonatal seizures^2^

***Case definition***

A *neonatal seizure* is defined as a transient electrographic change in the brain due to an abnormal, excessive or synchronous neuronal activity either with the occurrence of clinical signs (electro-clinical) or without them (electrographic-only), in the first 28 days of life in full-term infants. In the preterm infants (born <37 weeks of gestation), this definition applies up to 44 weeks of post menstrual age (PMA), considering the pattern of brain maturation.

Seizures confirmed by conventional EEG (cEEG) with or without clinical manifestations represent the most accurate concept of neonatal seizures; cEEG is considered the gold standard for neonatal seizure diagnosis (Level 1 – “definite” diagnosis). Ictal EEG refers to the epileptiform activity seen during a seizure in contrast to interictal discharges seen between seizures which are not diagnostic in neonates. Concomitant video recording is helpful although not a necessity and may be replaced by clinical observation during the EEG to determine a clinical-electrographic correlation.

Amplitude-integrated EEG (aEEG) or cerebral function monitoring can be a useful instrument but is less accurate than cEEG (see Section 1.1.7). The identification of seizures on the aEEG is considered a “probable” diagnosis of neonatal seizure (Level 2a).

As mentioned above, the clinical diagnosis of neonatal seizures is challenging and without EEG it is difficult to differentiate seizure from physiological or abnormal, but non-epileptic, movements (see Section 1.1.8). However, two seizure types are highly indicative of epileptic seizures, specifically focal tonic seizures (focal sustained stiffening/sustained increase in muscle contraction lasting a few seconds to minutes) or focal clonic (regularly rhythmic jerking, that involves the same muscle groups), which are not influenced by manual restraint [77]. Therefore, these seizure types also can be considered “probable seizures” (Level 2b) in the absence of a confirmation EEG, if observed by experienced medical personnel (a history of such events is not considered sufficient). The term “experienced medical personnel” refers to who routinely care for neonates and are familiar with the clinical presentation of neonatal seizures through training or clinical practice. Ideally this is a physician (not restricted to neonatology or neurology specialists), but in different settings also other professionals (such as advanced care provider, nurse, or individual such as midwife, health care worker) could diagnose “probable or possible seizures”, depending of their specific training in neonatal care.

As discussed in Section 1.1.11, neonatal seizure types also include other motor or non-motor manifestations such as myoclonic jerks, epileptic spasms, automatisms, autonomic changes and behavioral arrest. Based only on clinical observation (without EEG confirmation) it is not possible to label these manifestations as definite neonatal seizures, however, they can be considered “possible” seizure (Level 3), if observed by experienced medical personnel (a history of such events is not considered sufficient). Generalized tonic events and bilateral hypermotor events are usually non-epileptic.

For further information on clinical manifestations and definitions of seizure types and epilepsy syndromes see https://www.epilepsydiagnosis.org/index.html.

**LEVELS OF CERTAINTY**

**For All Levels of Diagnostic Certainty**

Age 0–28 days in a full-term infant

OR

Postmenstrual age of <44 weeks in a preterm infant (born <37 weeks of gestation)

**Level 1 of diagnostic certainty**

**Figure:**
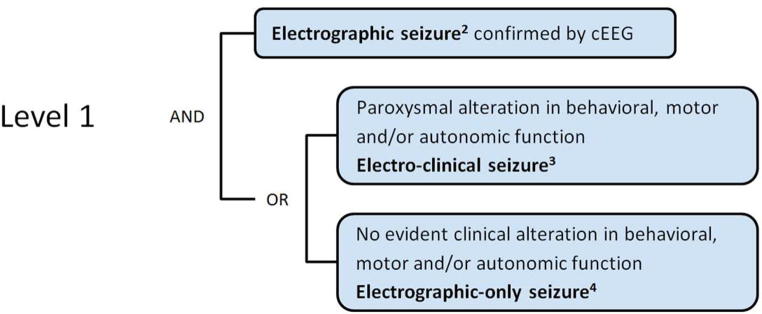

**Level 2 of diagnostic certainty**

**Figure:**
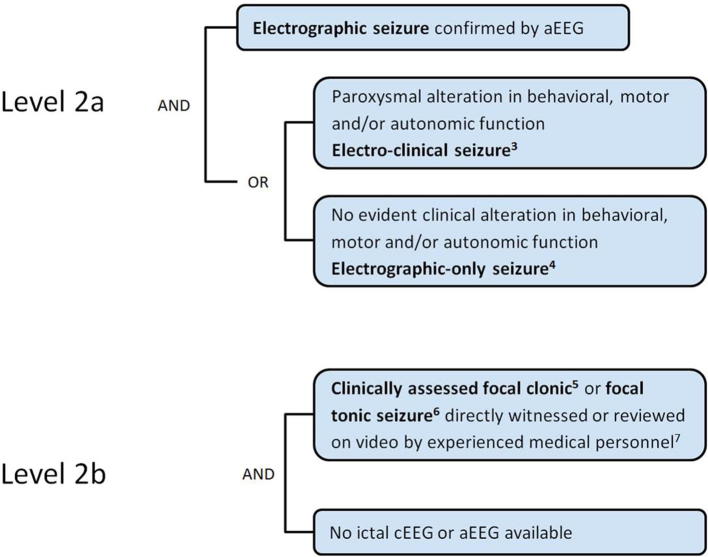

**Level 3 of diagnostic certainty**

**Figure:**
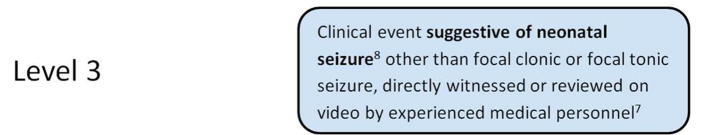

**Level 4**

**Figure:**
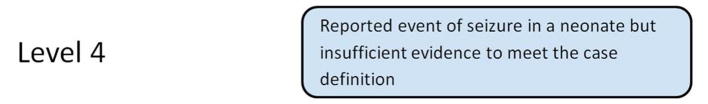

**Level 5**

**Figure:**
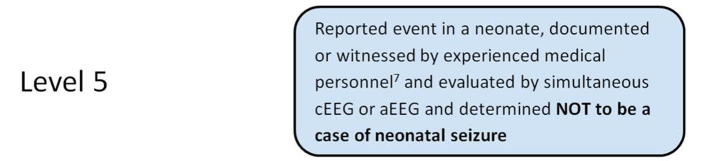

Notes for Levels of Certainty

^2^sudden, abnormal EEG event characterized by repetitive and evolving pattern (in frequency, voltage, morphology, or location)

^3^seizure confirmed with EEG and with clear clinical manifestation

^4^seizure confirmed with EEG without clear clinical manifestation

^5^regularly rhythmic jerking, that involves the same muscle groups and not influenced by manual restraint

^6^focal sustained stiffening/sustained increase in muscle contraction lasting a few seconds to minutes and not influenced by manual restraint

^7^someone who routinely cares for neonates and is familiar with the clinical presentation of neonatal seizures through training or clinical practice. Ideally this is a physician (not restricted to neonatology or neurology specialists), but in different settings also other professionals (such as advanced care provider, nurse, or individual such as midwife, health care worker) could diagnose “probable or possible seizures”, depending of their specific training in neonatal care

^8^such as myoclonic, epileptic spasm, automatism, autonomic changes, behavioral arrest, but non-seizure events cannot be excluded without EEG [79]

## 3 Guidelines for data collection, analysis and presentation of neonatal seizures

It was the consensus of the Brighton Collaboration *Neonatal Seizures Working Group* to recommend the following guidelines to enable meaningful and standardized collection, analysis, and presentation of information about neonatal seizures. However, the implementation of all guidelines might not be possible in all settings. The availability of information may vary depending upon resources, geographical region, and whether the source of information is a prospective clinical trial, a post-marketing surveillance or epidemiological study, or an individual sporadic report of neonatal seizures. Also, these guidelines have been developed by this working group for guidance only and are not to be considered a mandatory requirement for data collection, analysis, or presentation.

### 3.1 Data collection

These guidelines represent a desirable standard for the collection of data on neonatal seizures following maternal immunization to allow for comparability of data and are recommended as an addition to data collected for the specific study question and setting. The guidelines are not specifically intended to guide the primary reporting of neonatal seizures to a surveillance system or study monitor, but they could potentially be adapted for these purposes. Investigators developing a data collection tool based on these data collection guidelines also need to refer to the criteria in the case definition, which are not repeated in these guidelines.

Guidelines numbered below have been developed to address data elements for the collection of adverse event information as specified in general drug safety guidelines by the International Conference on Harmonization of Technical Requirements for Registration of Pharmaceuticals for Human Use, and the form for reporting of drug adverse events by the Council for International Organizations of Medical Sciences. These data elements include an identifiable reporter and patient, one or more prior maternal immunization, and a detailed description of the adverse event, in this case, of neonatal seizures following maternal immunization. The additional guidelines have been developed as guidance for the collection of additional information to allow for a more comprehensive understanding of neonatal seizures following maternal immunization.

#### 3.1.1 Source of information/reporter

For all cases and/or all study participants (including mothers and infants, as appropriate), the following information should be recorded:

(1) Date of report.
(2) Name and contact information of person reporting^10^ and/or diagnosing the neonatal seizures as specified by country-specific data protection law.
(3) Name and contact information of the investigator responsible for the subject, as applicable.
(4) Relation to the patient (e.g., clinician, nurse, family member [indicate relationship], other).

#### 3.1.2 Vaccinee/Control

##### 3.1.2.1 Demographics

For all cases and/or all study participants (including mothers and infants as appropriate), the following information should be recorded:

(5) Case/study participant identifiers (e.g. first name initial followed by last name initial) or code (or in accordance with country-specific data protection laws).
(6) Date of birth, age, and sex.
(7) For neonates: gestational age and birth weight, twin status.

##### 3.1.2.2 Clinical and immunization history

For all cases and/or all study participants (including mothers and infants as appropriate), the following information should be recorded:

(8) Past and current gynecological/obstetric history, medical history, including hospitalizations, underlying diseases/disorders, pre- immunization signs and symptoms including identification of indicators for, or the absence of, a history of allergy or other reactions to vaccines, vaccine components or medications; food allergy; allergic rhinitis; eczema; asthma. Any family history of seizure, neonatal/infant death (sibling), or congenital/genetic conditions should be recorded.
(9) Any medication history (other than treatment for the event described) prior to, during, and after maternal immunization during pregnancy including prescription and non-prescription medication as well as medication or treatment with long half-life or long-term effect. (e.g. immunoglobulins, blood transfusion and immunosuppressant).
(10) Maternal and infant immunization history (i.e. previous immunizations and any adverse event following immunization (AEFI), in particular occurrence of neonatal seizures after a previous immunization).

#### 3.1.3 Details of maternal and infant immunizations

For all cases and/or all study participants (including mothers and infants as appropriate), the following information should be recorded:

(11) Date and time of maternal and infant immunization(s).
(12) Description of vaccine(s) (name of vaccine, manufacturer, lot number, dose (e.g. 0.25 mL, 0.5 mL, etc.) and number of dose if part of a series of immunization s against the same disease).
(13) The anatomical sites (including left or right side) of all immunizations (e.g. vaccine A in proximal left lateral thigh, vaccine B in left deltoid).
(14) Route and method of administration (e.g. oral, intramuscular, intradermal, subcutaneous, and needle-free [including type and size], and vaccine vial [used/open vial or new vial] other injection devices).
(15) Needle length and gauge.

#### 3.1.4 The adverse event

(16) For all cases at any level of diagnostic certainty and for reported events with insufficient evidence, the criteria fulfilled to meet the case definition should be recorded.

Specifically document:

(17) Clinical description of signs and symptoms of neonatal seizures, seizure type [79] and if there was medical confirmation of the event (i.e. patient seen by appropriate health care provider^7^, and/or testing performed).
(18) Date/time of onset^11^, first observation^12^ and diagnosis^13^, duration and frequency of seizures (seizures/hour or seizures/day), last seizure^14^ and final outcome^15^.
(19) Concurrent signs, symptoms, and diseases.
  • Measurement/testing [89].
  • Minimum EEG standards for cEEG are described in the American Clinical Neurophysiology Society (ACNS) guidelines [73], [89].
  • Minimum aEEG standards are described by de Vries and Hellström-Westas (https://doi.org/10.1136/adc.2004.062745) [90] and also in the American Clinical Neurophysiology Society (ACNS) guidelines (https://www.acns.org/UserFiles/file/Guideline5-MinimumTechnicalStandardsforPediatricEEG_v1.pdf) [73].
  • Details of EEG (Date, type, duration, quality)
  • Results of electrolytes, blood gas, and serum glucose, calcium, magnesium, bilirubin as well as complete blood count and blood culture.
  • Other investigations depend on clinical presentation, history and availability and may include lumbar puncture, urine culture and toxicology (maternal toxicology screen), screen for relevant congenital infections, metabolic screen, and genetic testing.
  • Ultrasound and neuroimaging (MRI or CT scan) if available.
(20) Treatment given for neonatal seizures, especially specify drug(s) and dosing.
(21) Outcome^15^ at last observation. Persistence beyond the neonatal period should be noted, ideally as late as 12–18 months.
(22) Objective clinical evidence supporting classification of the event as “serious” according to regulatory standards^16^.
(23) Maternal and infant exposures other than the maternal immunization, including those 24 h before and after immunization, and until delivery (e.g. food, medications, environmental, etc.) considered potentially relevant to the reported event.

#### 3.1.5 Miscellaneous/general

The duration of surveillance for neonatal seizures should be predefined based on the neonatal period (see case definition – up to 28 days in term and up to 44 PMA in preterm infants). Events with onset of seizures after this time are not considered neonatal seizures although it is recognized that seizures may persist (onset of epilepsy).

Biologic characteristics of the vaccine (e.g. live attenuated versus inactivated component vaccines), biologic characteristics of the vaccine-targeted disease, biologic characteristics of the vaccinee (e.g. nutrition, underlying disease like immune-depressing illness) are not considered relevant for the choice of the duration of the surveillance for neonatal seizures.

(24) The duration of follow-up reported during the surveillance period should be predefined likewise. It should aim to continue to resolution of the event.
(25) Methods of data collection should be consistent within and between study groups, if applicable.
(26) Follow-up of cases should attempt to verify and complete the information collected as outlined in data collection guidelines 1–23.
(27) Investigators of patients with neonatal seizures should provide guidance to reporters to optimize the quality and completeness of the information provided.
(28) Reports of neonatal seizures should be collected throughout the study period regardless of the time elapsed between maternal or infant immunization and the adverse event. If this is not feasible due to the study design, the study periods during which safety data are being collected should be clearly defined.

### 3.2 Data analysis

The following guidelines represent a desirable standard for analysis of data on neonatal seizures to allow for comparability of data and are recommended as an addition to data analyzed for the specific study question and setting.

(29) Reported events should be classified in one of the following five categories including the three levels of diagnostic certainty. Events that meet the case definition should be classified according to the levels of diagnostic certainty as specified in the case definition. Events that do not meet the case definition should be classified in the additional categories for analysis.
**Event classification in 5 categories**^17^
  **Event meets case definition**
    Level 1: Criteria as specified in the neonatal seizures case definition
    Level 2: Criteria as specified in the neonatal seizures case definition
    Level 3: Criteria as specified in the neonatal seizures case definition
  **Event does not meet case definition**
  ***Additional categories for analysis***
    Level 4: Reported neonatal seizures with insufficient evidence to meet the case definition^18^
    Level 5: Not a case of neonatal seizures^19^
(30) The interval between maternal immunization and reported neonatal seizures is defined as the date/time of maternal immunization to the date/time of onset^11^ of the first symptoms and/or signs consistent with the definition. Additionally, the occurrence of neonatal seizures in relation to the infant’s date of birth should be reported. If few cases are reported, the specific time course could be analyzed for each; for a large number of cases, data can be analyzed in the increments based on trimester of maternal immunization (see Table 6a).

**Table 6:** Reporting of time intervals. (a) Subjects with neonatal seizures in relation to trimester of maternal immunization. (b) Subjects with neonatal seizures in relation to date of birth (maternal vaccination received any time during pregnancy).

Interval	Number
**(a)**	
First trimester	
Second trimester	
Third trimester	
**TOTAL**	

**(b)**	
First 24 h of life (Day 1)	
First 96 h of life (Day 1–4)	
First week of life (Day 1–7)	
Weeks 2–4 of life (Day 8–28)	
**TOTAL**

Furthermore, it is useful to analyze time of onset of seizure because some etiologies have a definite time of onset. For preterm infants the age of onset is recorded as the corrected age and chronological age (Table 6b).

(31) The period of occurrence is defined as the interval between the date of onset of the first seizure consistent with the definition and the last seizure^14^ and/or final outcome^15^. If seizures persist beyond the neonatal period, this has to be noted. Whatever start and end are used, they should be used consistently within and across study groups.
(32) If more than one measurement of a particular criterion is taken and recorded, the value corresponding to the greatest magnitude of the adverse experience could be used as the basis for analysis. Analysis may also include other characteristics like qualitative patterns of criteria defining the event.
(33) The distribution of data (as numerator and denominator data) could be analyzed in predefined increments (e.g. measured values, times), where applicable. Increments specified above should be used. When only a small number of cases are presented, the respective values or time course can be presented individually.
(34) Data on neonatal seizures obtained from subjects born to mothers receiving a vaccine should be compared with those obtained from an appropriately selected and documented control group(s) to assess background rates of neonatal seizures in non-exposed populations and should be analyzed by study arm and dose where possible, e.g. in prospective clinical trials.

### 3.3 Data presentation

These guidelines represent a desirable standard for the presentation and publication of data on neonatal seizures following maternal immunization to allow for comparability of data and are recommended as an addition to data presented for the specific study question and setting. Additionally, it is recommended to refer to existing general guidelines for the presentation and publication of randomized controlled trials, systematic reviews, and meta-analyses of observational studies in epidemiology (e.g. statements of Consolidated Standards of Reporting Trials (CONSORT) [91], of Improving the quality of reports of meta-analyses of randomized controlled trials (QUORUM) [92], and of Meta-analysis Of Observational Studies in Epidemiology (MOOSE) [93], respectively).

(35) All reported events of neonatal seizures should be presented according to the categories listed in guideline 29 or other classification that is considered appropriate.
(36) Data on possible neonatal seizures events should be presented in accordance with data collection guidelines 1–23 and data analysis guidelines 29–34.
(37) Terms to describe neonatal seizures such as “low-grade”, “mild”, “moderate”, “high”, “severe” or “significant” are highly subjective, prone to wide interpretation, and should be avoided, unless clearly defined.
(38) Data should be presented with numerator and denominator (n/N) (and not only in percentages), if available.
(39) Although denominator data are usually not readily available for immunization safety surveillance, attempts should be made to identify approximate denominators. The source of the denominator data should be reported, and calculations of estimates be described (e.g. manufacturer data such as total doses distributed, reporting through Ministry of Health, coverage/population-based data, etc.). The incidence of cases in the study population should be presented and clearly identified as such in the text.
(40) If the distribution of data is skewed, median and range are usually the more appropriate statistical descriptors than a mean. However, the mean and standard deviation should also be provided.
(41) Any publication of data on neonatal seizures after maternal immunization should include a detailed description of the methods used for data collection and analysis as possible. It is essential to specify:
  • The study design;
  • The method, frequency and duration of monitoring for neonatal seizures;
  • The trial profile, indicating participant flow during a study including drop-outs and withdrawals to indicate the size and nature of the respective groups under investigation;
  • The type of surveillance (e.g. passive or active surveillance);
  • The characteristics of the surveillance system (e.g. population served, mode of report solicitation);
  • The search strategy in surveillance databases;
  • Comparison group(s), if used for analysis;
  • The instrument of data collection (e.g. standardized questionnaire, diary card, report form);
  • Whether the day of maternal immunization was considered “day one” or “day zero” in the analysis;
  • Whether the date of onset^2^ and/or the date of first observation^3^ and/or the date of diagnosis^4^ was used for analysis; and
  • Use of this case definition for neonatal seizures, in the abstract or methods section of a publication^20^.

Notes for guidelines

^10^If the reporting center is different from the vaccinating center, appropriate and timely communication of the adverse event should occur.

^11^The date and/or time of onset is defined as the time within the neonatal period when the first sign or symptom indicative of neonatal seizures occurred. This may only be possible to determine in retrospect.

^12^The date and/or time of first observation of the first sign or symptom indicative for neonatal seizures can be used if date/time of onset is not known.

^13^The date of diagnosis of an episode is the day within the neonatal period when the event met the case definition at any level.

^14^The end of the occurrence of neonatal seizures is defined as the time the subject no longer meets the case definition at the lowest level of the definition.

^15^E.g. recovery to pre-event immunization health status, spontaneous resolution, therapeutic intervention, persistence of the event, sequelae, death.

^16^An adverse event after immunization (AEFI) is defined as serious by international standards [94] if it meets one or more of the following criteria: (1) it results in death, (2) is life-threatening, (3) requires inpatient hospitalization or results in prolongation of existing hospitalization, (4) results in persistent or significant disability/incapacity, (5) is a congenital anomaly/birth defect, (6) is a medically important event or reaction.

^17^To determine the appropriate category, the user should first establish, whether a reported event meets the criteria for the lowest applicable level of diagnostic certainty, e.g. Level three. If the lowest applicable level of diagnostic certainty of the definition is met, and there is evidence that the criteria of the next higher level of diagnostic certainty are met, the event should be classified in the next category. This approach should be continued until the highest level of diagnostic certainty for a given event could be determined. If the lowest level of the case definition is not met, it should be ruled out that any of the higher levels of diagnostic certainty are met and the event should be classified in categories four or five. The highest possible level of classification should be recorded for each event.

^18^If the evidence available for an event is insufficient because information is missing, such an event should be categorized as “Reported neonatal seizures with insufficient evidence to meet the case definition”.

^19^An event does not meet the case definition if investigation reveals a negative finding of a necessary criterion (necessary condition) for diagnosis. Such an event should be rejected and classified as “Not a case of neonatal seizures”.

^20^Use of this document should preferably be referenced by referring to the respective link on the Brighton Collaboration website (http://www.brightoncollaboration.org).

## 4 Disclaimer

The findings, opinions and assertions contained in this consensus document are those of the individual scientific professional members of the working group. They do not necessarily represent the official positions of each participant’s organization (e.g., government, university, or corporation). Specifically, the findings and conclusions in this paper are those of the authors and do not necessarily represent the views of their respective institutions.

## Declaration of Competing Interest

The authors declared that there is no conflict of interest.

## Figures and Tables

**Figure:**
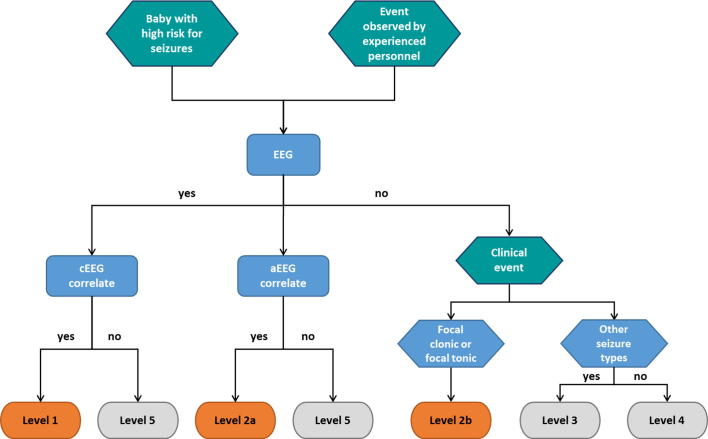


## Footnotes

2 The case definition should be applied when there is no clear alternative diagnosis for the reported event to account for the combination of symptoms.
