# Supplementary material for: Neonatal seizures: Case definition & guidelines for data collection, analysis, and presentation of immunization safety data
Source: Vaccine. 2019 Dec 10;37(52):7596–609. doi: 10.1016/j.vaccine.2019.05.031 (PMC6899436; doi:10.1016/j.vaccine.2019.05.031)
Supplement: Supplementary data 1 [file mmc1.docx]

# APPENDIX A: Tool to aid identification of appropriate level of diagnostic certainty for Neonatal Seizures

1. Is there experienced medical personnel available to evaluate the newborn?
   1. If yes 🡪 proceed to question 2.
   2. If no 🡪 STOP Neonatal seizures are not diagnosed.
2. Is there an adequate neurophysiological test available?
   1. If EEG 🡪 proceed to question 3.
   2. If aEEG 🡪 skip to question 4.
   3. If no 🡪 skip to question 5.
3. Are there clear electrographic seizures confirmed by EEG (≥ 10 sec) [[73](#_ENREF_73)]?
   1. If no events were captured during the EEG 🡪 skip to question 5.
   2. If typical events occurred during the EEG but were not associated with EEG correlate 🡪 STOP Neonatal seizures are not diagnosed.
   3. If yes, Gold standard 🡪 LOC 1.
      (to determine seizure type proceed to question 8).
4. Are there clear electrographic seizures confirmed by aEEG [[90](#_ENREF_90)]?
   1. If no 🡪 proceed to question 5.
   2. If yes 🡪 below gold standard 🡪 LOC 2a
      (to determine seizure type proceed to question 8).
5. Is the neonate treated with a muscle relaxant?
   1. If no 🡪 proceed to question 6.
   2. If yes 🡪 STOP Neonatal seizures are not diagnosed.
6. Has experienced medical personnel observed a clinically assessed focal clonic or focal tonic seizure (directly witnessed or documented on home video) as defined by Fisher et al. [[77](#_ENREF_77)]?
7. If no 🡪 proceed to question 7.
8. If yes 🡪 below gold standard 🡪 LOC 2b.
9. Has experienced medical personnel observed clinical events suggestive of epileptic seizures other than focal clonic or focal tonic seizures (directly witnessed or documented on home video)?
10. If no 🡪 STOP Neonatal seizures are not diagnosed.
11. If yes 🡪 well below gold standard 🡪 LOC 3.
12. Determine seizure type when seizures have been diagnosed according to LOC 1 or LOC 2
13. No evident clinical alteration in behavioral, motor and/or autonomic function 🡪 **Electrographic-only seizure.**
14. Paroxysmal alteration in behavioral, motor and/or autonomic function 🡪 **Electro-clinical seizure** (further seizures classification according to Fisher et al. [[77](#_ENREF_77)]).
