# Supplementary figures and images for: Neonatal seizures: Case definition & guidelines for data collection, analysis, and presentation of immunization safety data
Source: Vaccine. 2019 Dec 10;37(52):7596–609. doi: 10.1016/j.vaccine.2019.05.031 (PMC6899436; doi:10.1016/j.vaccine.2019.05.031)

**APPENDIX B: Flow chart**
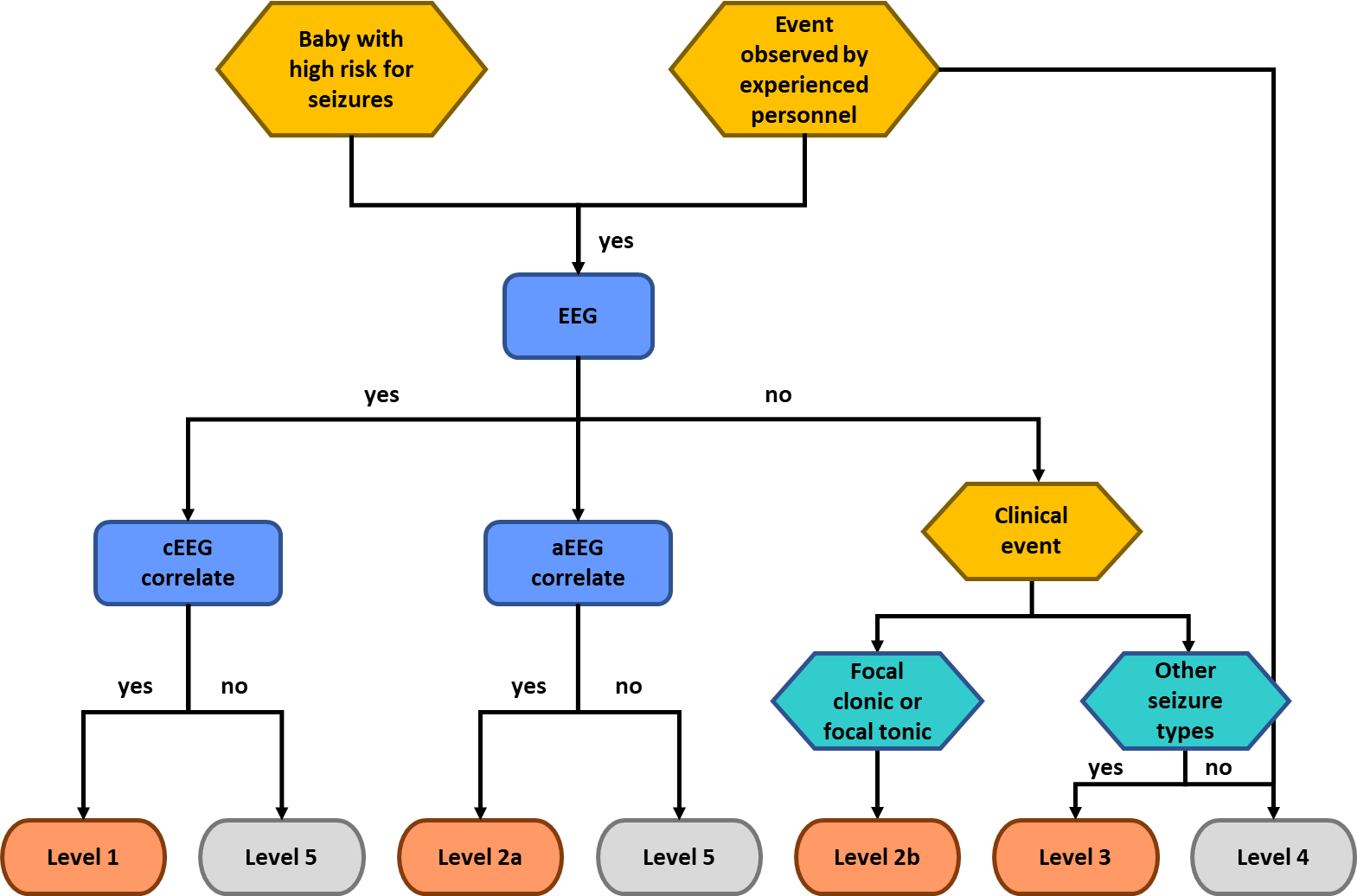

Supplement: Supplementary data 2 [file mmc2.docx]
